# Active transmembrane drug transport in microgravity: a validation study using an ABC transporter model

**DOI:** 10.12688/f1000research.4909.1

**Published:** 2014-08-21

**Authors:** Sergi Vaquer, Elisabet Cuyàs, Arnau Rabadán, Albert González, Felip Fenollosa, Rafael de la Torre

**Affiliations:** 1 Departament de Farmacologia Humana, Institut Municipal d’Investigació Mèdica de Barcelona (IMIM), Barcelona, 08003, Spain; 2 Corporació Sanitària i Universitària Parc Taulí, Sabadell, 08208, Spain; 3 Fundació Centre CIM, Barcelona, 08028, Spain

## Abstract

Microgravity has been shown to influence the expression of ABC (ATP-Binding Cassette) transporters in bacteria, fungi and mammals, but also to modify the activity of certain cellular components with structural and functional similarities to ABC transporters. Changes in activity of ABC transporters could lead to important metabolic disorders and undesired pharmacological effects during spaceflights. However, no current means exist to study the functionality of these transporters in microgravity. To this end, a Vesicular Transport Assay ^®^ (Solvo Biotechnology, Hungary) was adapted to evaluate multi-drug resistance-associated protein 2 (MRP2) trans-membrane estradiol-17-β-glucuronide (E17βG) transport activity, when activated by adenosine-tri-phosphate (ATP) during parabolic flights. Simple diffusion, ATP-independent transport and benzbromarone inhibition were also evaluated. A high accuracy engineering system was designed to perform, monitor and synchronize all procedures. Samples were analysed using a validated high sensitivity drug detection protocol. Experiments were performed in microgravity during parabolic flights, and compared to 1g on ground results using identical equipment and procedures in all cases. Our results revealed that sufficient equipment accuracy and analytical sensitivity were reached to detect transport activity in both gravitational conditions. Additionally, transport activity levels of on ground samples were within commercial transport standards, proving the validity of the methods and equipment used. MRP2 net transport activity was significantly reduced in microgravity, so was signal detected in simple diffusion samples. Ultra-structural changes induced by gravitational stress upon vesicle membranes or transporters could explain the current results, although alternative explanations are possible. Further research is needed to provide a conclusive answer in this regard. Nevertheless, the present validated technology opens new and interesting research lines in biology and human physiology with the potential for significant benefits for both space and terrestrial medicine.

## Introduction

Critical medical situations can occur in space missions ^1^. In such situations, adequate treatment may require the use of drugs with potential severe side effects. Pharmacokinetics and pharmacodynamics can be modified under weightlessness conditions by many factors ^2, 3^, which may lead to undesired pharmacological effects. These parameters should be assessed carefully in order to ensure safe use of medications in space. However, insufficient information exists in this regard in microgravity ^4, 5^ and proper empirical evaluation is not feasible due to the significant associated risks.

ABC (ATP-Binding Cassette) transporters are a large family of trans-membrane proteins widely present in many life forms, from bacteria to mammals. In humans, they play important roles in many physiological processes and in drug pharmacokinetics, pharmacodynamics, and drug-to-drug interactions of many currently used medications in oncology, emergency medicine, critical care and general practice ^6, 7^. Inhibition of these transporters has been correlated with clinically relevant metabolic disorders, drug overdosing, and drug toxicity ^7^. On the other hand, their overexpression can lead to treatment failure due to enhanced drug cell clearance and increased drug excretion from the body ^7^. Multi-drug resistance-associated protein 2 (MRP2) is a well-studied representative of the ABC transporters family, by which transport of glucuronide, sulphate and gluthatione-conjugated drugs is made against a concentration gradient requiring adenosine-tri-phosphate (ATP) hydrolysis ^8^.

Genetic expression of ABC transporters has been found to change in real and simulated microgravity conditions. Two independent studies evaluated gene expression pattern in two models of medically relevant species of microorganisms ( *Salmonella* sp. and *Candida* sp.) and found upregulation of certain ABC transporters genes during short duration spaceflight missions ^9, 10^. Similarly, results from a microgravity simulation study showed that significant variation of certain ABC transporter levels could be found in the liver and kidneys of a murine model ^11^. These changes could lead to a modified antimicrobial susceptibility in microgravity and to different pharmacokinetic characteristics of medications during spaceflight. Furthermore, recent reports suggest that microgravity can modify the activity of certain important cell components ^12– 14^, especially transmembrane proteins and ion channels ^15– 17^ with structural and functional similarities to ABC transporters. Changes in ABC transporters activity could lead to clinically significant metabolic disorders and potentially dangerous undesired pharmacological effects during spaceflight. However, there is no current technology that permits a functional evaluation of these cellular components in microgravity. Therefore, we developed a new approach combining the use of a currently available commercial assay, a high precision electromechanical system and an enhanced drug detection protocol to evaluate ABC transporters activity in microgravity conditions. A validation study of this new biotechnological approach is presented.

## Glossary

Microgravity: A condition in which there is very little net gravitational force, as of a free-falling object, an orbit, or interstellar space. In this text microgravity, weightlessness and zero gravity (0g) are considered equivalents.

Parabolic flight/parabolic flight campaign: To create a weightless environment, an especially prepared airplane flies in a long parabolic arc, in which two 2g periods are experienced while ascending (injection phase) and recovering (pull out phase) from the manoeuvre. In between these two periods, the aircraft enters into a free falling state, lasting approximately 22s, in which microgravity is obtained inside the cabin. A regular parabolic flight consists of 30 parabolas with a number of breaks. A parabolic flight campaign consists of three flights.

## Methods

This study was performed during the 51 ^st^ European Space Agency (ESA) parabolic flight campaign, under the auspices of the “Fly your thesis” programme, and was developed following the specifications and experimental requirements of ESA/Novespace parabolic flight campaigns ^18^.

### Samples and procedures

A modified, validated and commercial Vesicular Transport Assay ^®^ (Solvo Biotechnology, Hungary) was used to evaluate MRP2 transport activity of estradiol 17-β-glucuronide (E17βG) in microgravity during parabolic flights. A short overview of this assay is provided hereinafter and additional information can be found at the producer’s website ( www.solvo.hu). Recombinant baculovirus infected insect cells (Sf9 cells) are used to produce microscopic vesicles containing at least 12–15% of the selected human transporter protein following a standardized and validated production protocol owned by the producer ^7^. Vesicles are built allowing transport towards the interior of the vesicle. Once a reaction is triggered, the substrate (E17βG) is transported into vesicles and retained. Vesicles are filtrated using a fiberglass Milipore filter (< 1μm pore size) and eluted with pure methanol to retrieve substrate, which is later analysed. In the Vesicular Transport Assay ^®^, a base sample suspension is produced containing selected vesicles (50 μg of pure MRP2 transporter per sample) and an assay mix composed by E17βG, co-transporters and ions. Concentrations of the assay mix and vesicle quantities followed the instructions contained in the protocol provided by the vesicle producer, however volumes were adjusted to adapt to minimum volume requirements of our automated electromechanical system and to reduce the error induced by mechanical actuators. By modifying the aforementioned base suspension, four different groups of samples were generated to evaluate four different assay conditions as recommended by the producer ^19^. MRP2+ samples, containing a fully functional MRP2 transporter, were generated in order to evaluate the full transport capabilities of the transporter. NoATP samples were generated by using the same composition of MRP2+ samples but an ATP-free solution was injected instead at the moment of reaction start. These groups of samples permitted the evaluation of ATP-independent transport activity, known to be present in the ABC transporters family. Following vesicle producer recommendations, we also generated an additional group of samples to which benzbromarone 7.5 mM (BZM - specific non-competitive inhibitor) was added. In this set of samples, pharmacological inhibition capabilities in microgravity could be assessed. Finally, MRPdef negative control samples were generated from vesicles with mutated MRP-like transporters. These vesicles are provided by the producer and present no active transport capabilities. Therefore, they permit evaluation of simple diffusion of substrate across membranes. These samples were used as negative controls for transporter activity measurements (see supplementary material *Appendix 1. Chemical products* for a more detailed sample composition description). Confirmation of transporter activation requires the detection of net E17βG quantities in activated samples (MRP2+, noATP and BZM samples). Therefore, the signal from MRPdef samples was subtracted from the actual signal in each group of samples to obtain net transport activity. We performed a preliminary evaluation of the assay, which showed fast initial reaction speeds with most of E17βG being transported within few seconds. Samples were prepared in small syringes (Becton, Dickinson and Company, New Jersey, USA) containing 360 μl of the assay suspension (vesicles + assay mix), frozen immediately at -80°C and transported in dry ice to the parabolic flight campaign site. During the parabolic flight, the samples were kept in certified cold containers at 2–6°C before being used. The first five parabolas of each flight were excluded in order for the investigator to acclimatize to microgravity. The reaction was manually triggered 1s after the 6 ^th^ parabola injection phase by a precise servo-mechanic injection of 250 μl 0.2M ATP mix or ATP-free buffer depending on the sample group (MRP2+, BZM and MRPdef samples vs. noATP samples). The reaction was stopped automatically inducing a drastic temperature reduction and substrate dilution by applying 1.5 ml of washing mix at 2–6°C after 19s, still in microgravity. This procedure was repeated identically for each parabolic manoeuvre. Washing mix composition followed standard protocol specifications by the vesicle producer but volumes were adjusted to adapt to minimum volume requirements of our automated electromechanical system and to reduce the error induced by mechanical actuators. E17βG-filled vesicles were recovered by Millipore fibreglass filtration. Once on ground, pure methanol elution was performed for E17βG recovery and stabilization before transport. Standard *Escherichia coli* glucuronidase enzymatic reaction was later required for glucuronide removal from estradiol. Final estradiol concentration was measured by gas chromatography coupled to a mass spectrometry detection system (6890-GC/5973-MSD, Agilent Technologies, California, USA) using a validated methodology ^20^. Reference experiments were repeated on ground in 1g conditions using the same equipment, materials and procedures as those used during parabolic flights. The time lapse between sample stabilization with methanol and analysis was comparable in both cases. Sample analysis was performed using the same laboratory equipment and procedures. Results from 1g on ground reference experiments were compared with those obtained in microgravity conditions.

In order to evaluate vesicle microscopic structural integrity after gravitational stress, vesicles were exposed to 2g centrifugal forces in 60 consecutive 30s periods simulating a parabolic flight. Vesicles were compared to non-centrifuged controls at 200× under methylene blue tincture by experienced anatomo-pathologists (Pathology Department, Corporació Sanitària Universitària Parc Taulí. Sabadell, Spain). Qualitative analysis of shape and mean size estimation of vesicles was performed by experienced anathomo-pathologists, as well as a semi-quantitative vesicle number estimation using multiple 1 mm ^2^ sector partial counting method.

### Equipment

**Figure 1.**
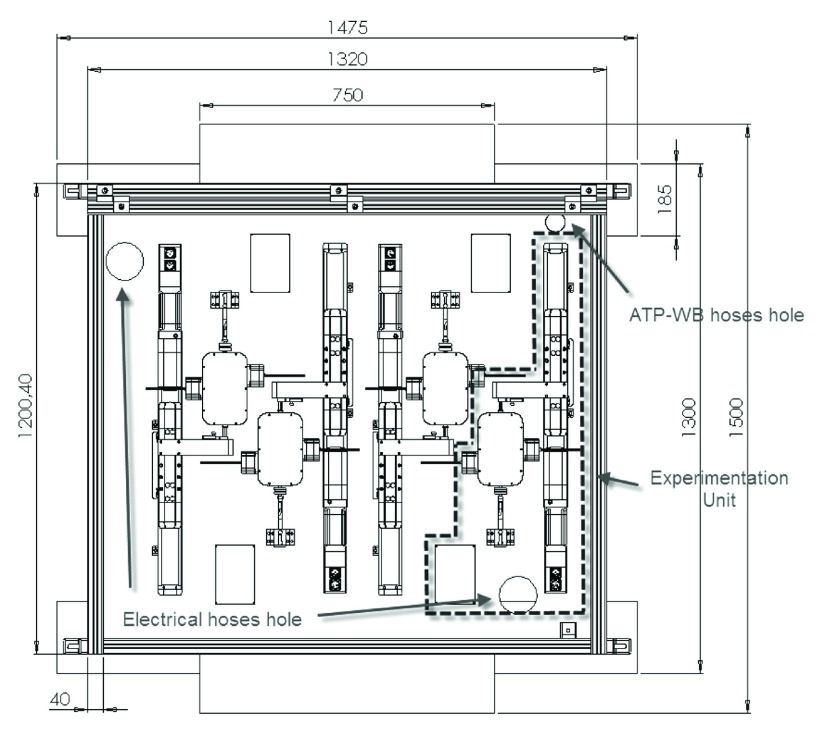
Main experimental rack. The designed electromechanical system was composed of three racks. In this figure a scheme of the main rack is presented, containing the four experimentation units. One of the four experimentation units is highlighted with a dashed line. Several electrical holes permitted connection to all electrical components in the main rack (sensors, actuators, linear engines, resistances). One ATP-WB hole permitted access to liquid conductions transporting ATP and Wash Buffer to each experimentation units. Lengths are expressed in mm.

**Figure 2.**
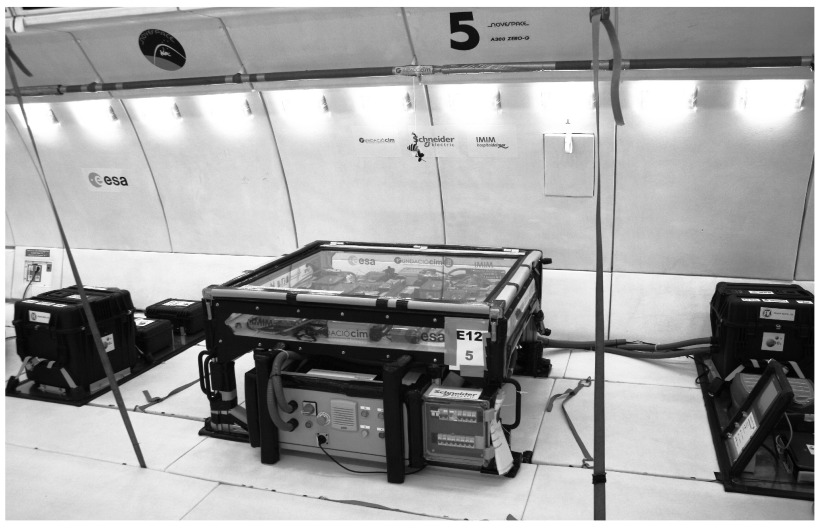
Final prototype configuration. This picture shows the final configuration of the experiment on board the parabolic aircraft. The three racks presented were, from left to right: storage rack, main rack, fluids and control rack. All principal electric components were placed in a sealed fire-proof cabinet underneath the main rack to protect the equipment from water contact in case of failure of the containment system.

An electro-mechanical engineering system was especially designed to undertake the required procedures in microgravity following the design and safety specifications for parabolic flights ( Figure 1 & Figure 2). Four experimentation units composed the prototype, one per each specific group of samples. Accurate fluid displacement was achieved implementing high precision component manufacturing and accurate linear engines (Schneider Electric, France). These engines presented 0.05 mm of displacement error, which represented a calculated 16 μl fluid displacement error (< 10% in our experimental setup). Sample temperature was strictly controlled using a dedicated thermal control system, which consisted of four preheating chambers to raise the sample temperature from 2–6°C to 37°C and four reaction chambers to maintain the temperature at 37.3°C during the reaction. In order to protect the vesicle integrity, 45°C was established as the maximum permissible temperature at syringe wall in preheating chambers given syringe radius and its thermal conductivity. Several temperature probes feedback a central computer, which maintained the temperatures at the required levels (Minco, Minneapolis, USA; TC-Direct, Madrid, Spain). This system also enabled cold storage of ATP and washing mix at 2–6°C in two certified isothermal boxes (Tecnisample, Barcelona, Spain). All procedures were controlled by an electrical and control system, which provided electrical power, actuator control, synchronization, monitoring, logging and a user-friendly interface (Telemecanique, France) (see supplementary material *Appendix 2. List of Components* for a more detailed description of equipment and components used).

### Statistical analysis

Results were analysed using SPSS v19.0 (International Business Machines, New York, United States of America). ANOVA test for multiple variable comparisons was performed to confirm differences in raw E17βG signal between the four sample groups in each gravitational condition. Bilateral Dunnett t post-hoc test was later used for comparing sample groups with MRPdef controls to confirm transporter activation. Provided equality of variances could not be assured and unbalanced size of groups being compared, non-parametric Mann-Witney U test was used to evaluate the differences between microgravity and 1g on ground samples. Statistical significance threshold was established at *p* < 0.05 bilaterally in all cases.

## Results

All four different assays presented expected E17βG signal profile in microgravity and on ground 1g conditions ( Table 1), being MRP2+ samples the most active and MRPdef the less active group of samples in all gravitational conditions. The ANOVA test revealed statistically significant signal differences between sample groups in each gravitational condition (on ground F = 4.95, *p* = 0.005 and microgravity F = 13.45, *p* < 0.001) and post-hoc Dunnett t test confirmed statistically significant differences between MRP2+ and MRPdef in both microgravity and 1g conditions ( Table 2). These results confirmed activation of the transporter in MRP2+ samples. MRP2 net transport activity of 1g on ground samples laid within commercial transport standards (4.61 ng in a range of 3.7 – 7.4 ng of E17βG in our experimental configuration), conversely microgravity samples suffered a 19-fold activity reduction with statistically significant differences of E17βG transported in all cases. The Mean activity differences between microgravity and 1g on ground samples were 10.3 ng of E17βG in MRP2+ samples (Mann-Witney U < 0.001; *p* < 0.001; CI 95% = 7.4 – 13.2 ng E17βG), 5.95 ng of E17βG in MRPdef samples (Mann-Witney U < 0.001; *p* < 0.001; CI 95% = 4 – 7.9 ng E17βG), 7.65 ng of E17βG in noATP samples (Mann-Witney U < 0.001; p < 0.001; CI 95% = 6.14 – 9.16 ng E17βG) and 9.12 ng of E17βG in BZM samples (Mann-Witney U < 0.001; *p* < 0.001; CI 95% = 7.9 – 10.3 ng E17βG).

**Table 1.** Detected E17βG signal in microgravity and 1g conditions.

	Sample	n	Mean*	SD	% ^†^
microgravity	MRP2+	26	0.49	0.17	100
	MRPdef	31	0.25	0.12	51
	noATP	25	0.29	0.14	60
	BZM	24	0.27	0.20	54
1g on ground	MRP2+	10	10.8	4.08	100
	MRPdef	12	6.20	3.08	57
	noATP	11	7.95	2.25	73
	BZM	9	9.38	1.59	86

MRP2+: fully activated MRP2 transporter. MRPdef: MRP-like defective transporter. noATP: without ATP. BZM: Inhibition by benzbromarone. * Mean E17βG signal in nanograms. ^†^ Percentage of activity.

**Table 2.** Net E17βG transport activity.

		Mean difference *	Dunnett t *p*	CI 95%
microgravity	MRP2+	0.24	< 0.001 ^†^	0.14 – 0.34
	noATP	0.05	0.565	-0.05 – 0.14
	BZM	0.02	0.952	-0.08 – 0.12
1g on ground	MRP2+	4.61	0.002 ^†^	1.54 – 7.69
	noATP	1.75	0.364	-1.25 – 4.74
	BZM	3.18	0.049 ^†^	0.01 – 6.35

MRP2+: fully activated MRP2 transporter. MRPdef: MRP-like defective transporter. noATP: without ATP. BZM: Inhibition by benzbromarone. CI 95%: confidence interval 95% * Mean difference is obtained by subtracting MRPdef signal from the signal detected in each group of samples. Net E17βG transported in nanograms. Denotes statistically significant

The use of consecutive 2g periods in order to simulate the effects of hypergravity present during a parabolic flight did not show differences in shape, size or number of vesicles between centrifuged vesicles and control samples. Aggregation of vesicles was not observed, neither macroscopically nor microscopically during this simulation. The thermal control system implemented in our prototype maintained the temperature of reaction chambers at 37.25°C (CI 95%: 37.14 – 37.34°C) during the whole duration of experiments and kept the sample syringe wall temperature below 45°C in preheating chambers in all cases.

Data of active transmembrane drug transport in microgravity Results from both microgravity and 1g on-ground experiments are presented (labelled _0g and _1g). In each gravitational condition four different assays were performed: fully activated transporter or “MRP2+”, simple diffusion/control or “MRPdef”, ATP-independent transport or “noATP”, BZM inhibition assay samples or “BZM”.

## Discussion

To our knowledge, this is the first validation study of a biotechnological approach aimed at evaluating human ABC transporters-mediated active transmembrane transport capabilities in microgravity. Despite the high signal variability expected, the precision attained by our electromechanical system and the enhanced sensibility of the detection protocol used, enabled us to detect net MRP2 transport activity in the short microgravity periods provided by parabolic flights. Furthermore, activation profile and signal levels detected in 1g on ground samples laid within expected commercial standards, confirming the validity of the equipment and procedures used.

The results obtained in microgravity samples showed a significant reduction in MRP2 transport activity, when compared to on ground 1g controls, but presented a comparable activation profile. In order to ensure reliability of these findings, vesicle stability during a parabolic flight was assessed on ground. Simulations revealed that vesicle microscopic structure could remain stable during parabolic manoeuvres, in spite of repeated hypergravity periods. Provided that materials, equipment, procedures and transportation means were equal for all samples in all gravitational conditions, we hypothesized that gravitational stress during parabolic flights may have exerted an effect upon the vesicle membrane or the transporter, preventing proper transport or retention of E17βG. However, such modification should occur at an ultra-structural level, out of reach of our microscopic evaluation. Unfortunately, given the lack of similar research precedents, an ultra-structural evaluation of samples was not planned and was not possible after analysis, since all microgravity samples had been eluted to retrieve E17βG. Therefore, the results of the present evaluation cannot provide a definitive explanation of the effect of microgravity upon ABC transporters-mediated active transmembrane transport. Notwithstanding the aforementioned limitation, we tried to elaborate a plausible hypothesis to explain the present observations.

Compelling evidence indicates that gravity is able to affect cellular and even molecular interactions in numerous life forms, such as in certain protozoa ^12, 14^, bacteria ^15^, plant cells ^13, 16, 17^ or mammals ^21– 23^. Enzymes from the cell membrane ^24^, cytoskeleton ^25^, cytosol ^26^ and nucleus ^24^ have been found to be sensible to gravitational changes. In principle, gravity is a weaker force compared to intermolecular bonds, polar-apolar interactions and van der Waals forces, therefore it is not clear yet how gravity could affect enzymatic reactions. However, changes in polar-apolar interactions and conformational changes of lipid structures occurring in microgravity could explain some observations ^24, 25^. Structures such as ion channels and trans-membrane signalling systems can be significantly affected by changes in physicochemical properties of the lipid bi-layer cell membrane. Similarly, the mechanism of action of ABC transporters, although still not fully unveiled, is highly dependent on transporter-membrane interactions ^28^, which play a principal role in transporter stabilization and substrate recognition. Interestingly, we were able to demonstrate a reduction of the signal detected in MRPdef controls, where only simple diffusion of substrate was possible. This finding could be explained by an impeded E17βG diffusion into vesicles, or by an increase in membrane permeability in microgravity, which would lead to increased E17βG leakage. In any case, the present observations are indicative of important structural changes in the lipid bi-layer in microgravity, which could be induced by modified polar-apolar interactions of lipid structures in microgravity, as suggested elsewhere ^28^. In this context, the interaction of ABC transporters with the membrane, substrate recognition capabilities and transporter activation could become significantly affected with the potential for relevant biological, physiological and medical consequences in microgravity. However, additional research is required to confirm the present hypothesis.

There are many potential applications of the presented technology, which can benefit both space and terrestrial medicine. Insufficient information exists on drug effects in space ^4, 5^ and is often based in very reduced observational studies ^30^ or in animal models ^31^. While clinical experience throughout spaceflight history has shown no critical metabolic alterations in astronauts, few medications have been studied from a pharmacological point of view in space. A reduction of transmembrane drug transport capabilities in microgravity could lead to drug accumulation and potentially toxic effects during spaceflight. However an increase in membrane permeability would lead to an enhanced drug clearance, increased absorption and significant changes in pharmacological proprieties of drugs. Although not conclusive, our results warrant additional and thorough evaluation of this and other human drug transportation systems in a more stable microgravity platform. To this end, this study describes a new method, based on *in vitro* analysis of human drug transmembrane transport capabilities, by which an approximation to drug effects in microgravity can be made. Similarly, reduced activity of certain ABC transporters has been associated with a number of serious medical conditions on Earth. On the other hand, enhanced activity of ABC transporters can decrease intestinal drug absorption, facilitate hepatic and renal drug excretion, modify drug distribution, and limit drug penetration to certain body tissues. These transporters are known to be responsible for a significant portion of the variability in treatment response to certain drugs used in emergency and critical care medicine ^6– 8^ and can cause multidrug resistance in a number of solid and hematological neoplasias, where overexpression of certain ABC transporters is present ^6, 7^. Further research derived from this study may help elucidate the intricate mechanism of action of ABC transporters and provide new information for developing more effective treatments for oncology and other medical specialities based on microgravity effects upon these transporters.

There are several limitations in this study. First, we designed a novel electromechanical system to allow the required biochemical reactions to be undertaken precisely and automatically in parabolic flights. This equipment had never been tested before in such conditions. However, results from 1g on ground control samples laid within commercial transport standards, proving functionality and validity of the research equipment and protocols used. Additionally, MRP2 net E17βG transport activity in microgravity fell within the detection range of our enhanced detection method, which confirmed that the required sensitivity was reached. Second, as previously addressed by Macarrone *et al.* ^28^, “microgravity is likely to favour the dispersion in water of less dense molecules such as lipids and the opposite is observed in hypergravity”. The contact surface available for ABC transporter exposition to drug-rich medium would be reduced, and transport capability would consequently be diminished, if vesicles precipitated during 2g phases in parabolic flights. Furthermore, vesicle structural integrity has never been evaluated in variable gravitational conditions. Nevertheless, no macroscopic precipitation of samples was observed in-flight due to combined effects of aircraft vibration and the ATP turbulent injection jet. On-ground hypergravity simulation data suggested that vesicles remained stable and did not aggregate after repeated 2g periods. However ultrastructural alterations affecting the membrane or the transporter could still be possible and were not evaluated. Further research should focus on performing a detailed ultra-structural evaluation of vesicles and transporters in a more stable microgravity platform and use whole human cells to provide a more conclusive answer on the potential physiological and clinical effects of our findings. Other factors were also considered as possible disruptors of results and were evaluated. The temperature was strictly maintained within a narrow margin during experiments but aircraft pressure was reduced in flight (800 hPa in flight). However, pressure variations were not expected to cause any modification in sample composition nor in transport activity in our experimental setup.

In conclusion, we validated a new methodology for evaluating ABC transporters activity in microgravity during parabolic flights. Our results demonstrated that despite time constrains, the combination of an adapted commercial assay, a highly accurate electromechanical system and an enhanced drug detection protocol can provide enough accuracy, reproducibility and sensitivity to detect transport activity in microgravity. A significant decrease in transport activity of fully activated samples and a reduction of signal detected in negative controls could be observed in microgravity. While the present results are insufficient for drawing conclusive explanations to these observations, we hypothesize that altered polar-apolar interactions induced by gravitational stress during parabolic manoeuvres affected vesicle’s lipid bi-layer membrane, eventually changing its physicochemical properties, limited simple diffusion phenomena and impaired transporter - membrane interaction. However, alternative explanations are possible. Therefore, additional studies will be required to assess membrane and transporter ultra-structures in microgravity, and to confirm these results. The novel methodological approach presented here opens new and interesting research lines in biology, microbiology and human physiology with the potential for significant benefits for both space and terrestrial medicine.

## Data availability

Dataset 1. Data of active transmembrane drug transport in microgravity, 10.5256/f1000research.4909.d34169 ^32^

## Figures and Tables

**Table:** 

Products used for in-flight operations
1.7M Tris (Tris[hydroxymethyl] aminomethane) Water based Dilution
0.1 M MOPS-Tris (Solution)
300 mM Glutathione (GSH)
DMSO. Dimethyl sulfoxide
7.5 mM E17βG in DMSO
0.2 M Mg-ATP (ATP, MgCl _2_, Tris)
0’1M MgCl _2_
0.14 M KCl
1 M KCl
7’5 mM Benzbromarone in DMSO
MRP2 Vesicles
Assay Mix (solution): 0.1M MOPS-Tri (50% of the solution), 0.14M KCl (50% of the solution), 0.1 MgCl _2_ (<1% of the solution)

**Table:** 

Solutions used for in-flight operations
Washing Mix: 0.1 MOPS-Tris (40% of the solution), 1M KCl (2,5% of the solution), Milli-Q Water (47,5% of the solution)
Syringes: MRP2/Control vesicles (<1%) + Assay Mix (97%) + Benzbromarone (1%) + GSH (<1%) + DMSO (1%) + Estradiol (<1%)
Assay Mix (solution): 0.1M MOPS-Tris (50% of the solution), 0.14M KCl (50% of the solution), 0.1 MgCl _2_ (<1% of the solution)
0.2 M Mg-ATP (ATP, MgCl _2_, Tris)

**Table:** 

Id	Company	Ref.	Description
Jx	BD	300910	Reaction syringe (3 ml)
J1	BD	300910	Reaction syringe (3 ml)
J2	BD	300912	ATP/Assay Mix syringe (60 ml)
J3	BD	300865	Washing mix syringe (60 ml)
J4	BD	300865	Washing mix syringe (60 ml)
J5	BD	300865	Waste syringe (60 ml)
J6	BD	300865	Waste syringe (60 ml)
J7	BD	300865	Waste syringe (60 ml)
Anti-leak diaphragm valve	BD	385100	Anti-leak valve
V1	BD	394600	3 ways valve
V2	BD	394600	3 ways valve
Tube PVC	BD	397251	PVC line extension

BD: Becton, Dickinson and Company

**Table:** 

Id	Company	Ref.	Description
TMP1-8	TC-Direct	410-215	Temperature probe
R1-4	Minco	HK5407R98.1L12A	Resistance heating
R5-8	Minco	HK5591R91.4L12A	Resistance heating
	Minco	#20 TAPE 6FT	Self-fusing silicone tape
FU1-4	RS components	176-9132	Resettable thermal fuse 84°C
FU5-8	RS components	176-9148	Resettable thermal fuse 72°C

**Table:** 

Id	Company	Ref.	Description
Polyurethane box	Tecnisample	ISOPUR 13	Contains 1 ATP syringe and 8 Washing mix syringes
Polystyrene box	Tecnisample	N/A	Contains 100 new sample syringes
Cold Accumulators	Tecnisample	N/A	Rigid plastic water accumulators

**Table:** 

Id	Company	Ref.	Description
PMAFLEX tube	PMA AG	CYLG-48B	Tube which guarantees no leaks and two isolation level between ATP+WB box and polycarbonate sealed box.
Connector	PMA AG	BVID-M508GT	Accessory to connect PMAFLEX tube with ATP+WB box and polycarbonate sealed box.
Connector	PMA AG	GMN-M50	Nut of BVID-M508GT
New syringes box	Peli	0340-001-110-E01	Case with a 67 IP protection level.
Used syringes box	Peli	1450-001-110-E01	Case with a 67 IP protection level.
Used filters box	Peli	1450-001-110-E01	Case with a 67 IP protection level.
ATP+WB box	Peli	0340-001-110-E01	Case with a 67 IP protection level.

**Table:** 

Id	Company	Ref.	Description
QS1	Telemecanique	VCF01	Isolating switch 20A
QF1	Merlin Guerin	C60N-11933	General breaker switch 2 Phases 7,5A
F1	Merlin Guerin	ID23523	General dif. switch 2 Phases 25A 30mA
QF2-14	Merlin Guerin	DPN-21555	Breaker switch 1 Phases 6A
FA1	Telemecanique	ABL-8FEQ24200	Power unit 20A 24Vdc
PT1	Telemecanique	XBT-GT4330	Touch screen
PLC 1	Telemecanique	BMX-XBP0800	Rack 8 positions
	Telemecanique	ABE7 CPA412	Fast connector
	Telemecanique	ABE7 H16R20	Fast connector
	Telemecanique	ABE7 R16T212	Fast connector
	Telemecanique	BMX-CPS2000	Power unit CPU 20W
	Telemecanique	BMX-P342010	CPU
	Telemecanique	BMX-DDI3202	Digital in card
	Telemecanique	BMX-DDO3202	Digital out card
	Telemecanique	BMX-DDO3202	Digital out card
	Telemecanique	BMX-ART0414	Thermocouple input card
	Telemecanique	BMX-ART0414	Thermocouple input card
SBE	Telemecanique	XB5-AS8445	General Emergency button
SB1	Telemecanique	XB5-AA42	Push Rearmament
KA1	Telemecanique	LC1DT20P7	Power relay (220VAC)
MLIN1	Schneider	SER-36103L75	Linear actuator
MLIN2	Schneider	SER-36103L75	Linear actuator
MLIN3	Schneider	SER-36103L75	Linear actuator
MLIN4	Schneider	SER-36103L75	Linear actuator
LXM05.1	Schneider	LXM05AD10M2	Servodrivers
LXM05.2	Schneider	LXM05AD10M2	Servodrivers
LXM05.3	Schneider	LXM05AD10M2	Servodrivers
LXM05.4	Schneider	LXM05AD10M2	Servodrivers
ST1	Schneider	BRS364H	Stepper motor
CST1	Schneider	SD3 15	Controller stepper motor
ST2	Schneider	BRS364H	Stepper motor
CST2	Schneider	SD3 15	Controller stepper motor
ST3	Schneider	BRS364H	Stepper motor
CST3	Schneider	SD3 15	Controller stepper motor
ST4	Schneider	BRS364H	Stepper motor
CST4	Schneider	SD3 15	Controller stepper motor
ST5	Schneider	BRS364H	Stepper motor
CST5	Schneider	SD3 15	Controller stepper motor
ST6	Schneider	BRS364H	Stepper motor
CST6	Schneider	SD3 15	Controller stepper motor
ST7	Schneider	BRS364H	Stepper motor
CST7	Schneider	SD3 15	Controller stepper motor
ST8	Schneider	BRS364H	Stepper motor
CST8	Schneider	SD3 15	Controller stepper motor
TMP1-8	TC-Direct	408–053	Temperature probe
R1-4	Minco	HK5407R98.1L12A	Heating resistance
R5-8	Minco	HK5591R91.4L12A	Heating resistance
E1-12	Schneider	Xs1N08PA349	Sensors 10A38VCC 2MM
	Weidmüller	WDK 2.5	Terminal block
	Himel	PLA-1573KT	Electrical box
	Himel	PMR157	Electrical plate
	Himel	S27 DL/24	Auxiliar electrical box
	Himel	PF-27	Fixing to auxiliar electrical box
	Himel	IBS 1912/8	Terminal electrical box (*2)
	Himel	PMDP 2015	Terminal electrical plate (*2)
PC	BENQ	R53U-S07- PM740C8UGH6	Laptop
	Himel	VF38	Ventilator (Refrigeration unit)
BTN	Telemecanique	XB4-BW33B5	Green light push button “START”
BTN	Telemecanique	XB4-BW36B5	Blue light push button “ARM”
BTN	Telemecanique	XB4-BW35B5	Yellow light push button “STOP”
BTN	Telemecanique	XB4-BW34B5	Red light push button “HALT”
	PMA	CYLG-29S	Plastic tube
	PMA	BVID-M329GT	Connector tube
	PMA	GMN-M32	Nut
	PMA	SVH-29-010	Clamps
	INTERFLEX	012577	Gland
	INTERFLEX	011277	Gland
	UNEX	60.40.77	Gutters
	UNEX	60.60.77	Gutters
	ILME	CNF16 CNM16	Harting connector
	ILME	MHO32L CHI32LS	Harting connector
	Schneider	DF2 CN08	Fast fuse 8A
	Schneider	DF6 AB10	Carrier fuse
FU1-4	RS	176-9148	Resettable thermal fuse 84°C
FU5-8	RS	176-9132	Resettable thermal fuse 72°C
KA2-9	Finder	Type 40.52	Relays
			220V female plug
	RS	327-131	220V male plug

## Appendix

### List of components

**Fluid System**

**Heating System**

**Cooling System**

**Containment System**

**Electrical System**
