# Supplementary material for: Active transmembrane drug transport in microgravity: a validation study using an ABC transporter model
Source: F1000Res. 2014 Aug 21;3:201. [Version 1] doi: 10.12688/f1000research.4909.1 (PMC4264636; doi:10.12688/f1000research.4909.1)
Supplement: Peer review history

# Peer review: Referee response for version 1

**DOI:** 10.5256/f1000research.5243.r6930

**Published:** 2014-12-05

**Authors:** Steven Rees

**Affiliations:** 1 Department of Pharmacology, University of California, San Diego, La Jolla, CA, USA

This paper is an interesting contribution to the study of membrane transporter activity in microgravity. The authors discuss in detail the problems not addressed by the study, and important future studies for further validation. This is an excellent addition to the literature, and should be indexed.

- Introduction was a concise and accurate overview of ABC transporters and microgravity simulations.
- Good use of controls, including consideration of differential permeability of vesicles themselves due to microgravity
- No control with BZM/deformed MPR2 to see differences in noncompetitive inhibition (see if there’s activity in negative control vesicles (MRP2def) to see if relevant, particularly for the point about inherent changes in vesicle permeability mentioned in Discussion).
- Vesicle integrity was confirmed with 2g on-ground simulated flight campaign study (why not just run study at 1g alongside samples to eliminate confounding variables?). Also, is it apparent that the 2g on-ground study truly replicates the experimental flight campaign?
- Table 2: NoATP mean diference, microgravity, is 0.04, not 0.05 (according to Table 1). Same mistake with 1g on ground MRP2+, though this is most likely due to rounding.
- Following Table 2’s purpose, comparing relative difference (dividing microgravity and 1g mean differences by MRP2def) would have made data more comparable, given stark differences in means presented in Table 1.
- Data seem to have high variance, despite precision provided by the in-flight device. Comparison of various flight results in groups would hint at potential time-based variance or subtle differences in parabolic flight that could have contributed to these results (repeated flights, to achieve true replicates over pseudoreplicates, could have also aided in this, though may be impractical given predicted high cost of this single flight campaign).
- No data presented on vesicle integrity outside of discussion in Results; even if not significant, would have been useful.
- It is not clear that microgravity causes an increase in vesicle permeability given the quite reduced transport of E17BG in microgravity, as suggested in the discussion, though the ratio of E17BG transport in microgravity is higher than that of 1g transport. Future studies looking into this would be useful.
- More information on parabolic flight campaign would have been useful, not only for reproducibility but given the novelty of this procedure. 
- Studying differential effects of microgravity on mammalian versus insect vesicles would be an important follow-up investigation, as suggested by the authors.

I have read this submission. I believe that I have an appropriate level of expertise to confirm that it is of an acceptable scientific standard.

# Peer review: Referee response for version 1

**DOI:** 10.5256/f1000research.5243.r5908

**Published:** 2014-08-28

**Authors:** Thais Russomano

**Affiliations:** 1 Faculdade de Medicina, Centro de Microgravidade/FENG-PUCRS, Porto Alegre, Brazil

It is a well written article and deserves to be indexed.

My comments and suggestions are:

- Abstract should explain the method better, including more details of controls on Earth. It should contain some data (numerical data with the most important statistical analysis)

Introduction is good.

Methods

- Samples and procedures: I suggest explaining the phases of the parabola and their duration. I also believe that it would be good to clarify how many parabolas were used for the tests.
- Figures are ok, although figure 2 does not add much to the study itself, unless some explanation is added to the figure and its caption.

Results

- I believe that figures could show the microG changes better
- If figures are kept, please correct first line on Table 1 (data is misplaced)
- I think it should be 1G (not just in results but throughout the text), and then ground should be deleted (it is a pleonasm in this case)

Discussion

- It is very good, clear, and consistent

Conclusion

- It could be shortened a bit.

It was very good to have considered and presented the limitations of the study.

I have read this submission. I believe that I have an appropriate level of expertise to confirm that it is of an acceptable scientific standard.
